# Anatomical Evaluation of the Foramen Magnum on Cone-Beam Computed Tomography Images and Review of Literature

**DOI:** 10.7759/cureus.19385

**Published:** 2021-11-08

**Authors:** İlhan Bahşi, Saliha Seda Adanır, Mustafa Orhan, Piraye Kervancıoğlu, Zeynep Simgül Büyükbeşe, Orhan Beger, Eda Didem Yalçın

**Affiliations:** 1 Department of Anatomy, Gaziantep University, Gaziantep, TUR; 2 School of Medicine, Gaziantep University, Gaziantep, TUR; 3 Department of Dentomaxillofacial Radiology, Dokuz Eylül University, İzmir, TUR

**Keywords:** morphometry, morphology, skull base, surgical anatomy, foramen magnum

## Abstract

Background and objective

The morphology and morphometry of the foramen magnum (FM), which provides a passageway to vital neurological structures that relay information to and from the brain and spinal cord, are significant for many surgical approaches and applications. It was aimed to investigate the morphometric and morphological features of the FM on the cone-beam computed tomography (CBCT) images and to review the literature in detail.

Methods

CBCT images of 400 individuals (200 males, 200 females, aged between 18 and 65) were evaluated by Planmeca Romexis Viewer. The length, width, perimeter, and area of the FM were examined and samples were classified according to shape. Also, the FM index (FMI) was calculated.

Results

The mean values of the length, width, and perimeter were found to be 36.75±2.50 mm, 32.55±2.93 mm, and 108.35±7.50 mm, respectively. The area of FM was found to be 941.81±128.26 mm^2^, 946.83±127.39 mm^2^, and 895.76±123.50 mm^2^ with Planmeca Romexis Viewer, Radinsky formula, and Teixeira formula, respectively. All parameters were significantly larger in males than females. There was no correlation between age and these parameters. Also, seven shapes were determined for FM, and the most common shape was oval. FM index was evaluated according to the Martin and Saller classification. It was found that 16.5% of the cases belonged to the narrow, 16% belonged to the medium, and 67.5% to the large FM index. There was no correlation between age and FM index.

Conclusion

Morphometric and morphological features of the FM located in the craniovertebral junction (CVJ), which is a highly complex area, are variable. Surgical procedures and approaches in this region are essential due to the high mortality rate. For this reason, anatomical structures in these regions should be well known before surgery. The quantitative data presented in this study, which made a detailed literature comparison, may assist in surgical procedures around the FM and the planning of these procedures.

## Introduction

The foramen magnum (FM) is the most remarkable structure located in the occipital bone of the skull. Vital neurological structures which relay information to and from the brain and spinal cord pass via FM. It lies in an anteromedial position and leads into the posterior cranial fossa [1]. FM also provides the transition of accessory nerve, vertebral artery, anterior spinal artery, and posterior spinal artery. Also, the tectorial membrane and apical ligament pass through it to attach to the margin of the FM [2]. Zdilla et al. [3] stated that the studies of the FM shape had been performed with ambiguous and subjective terminology that varies between previous studies. The classic anatomy and neurosurgery books stated that FM is oval and wider behind, with its greatest diameter being anteroposterior [1]. However, there are many studies that reported FM may be tetragonal, round, egg-shaped, hexagonal, pentagonal, and irregular [2,4,5]. Also, Chethan et al. [4] stated that the existence of some correlation between the shape of the FM and ancestry of an individual has been reported.

On the other hand, the high rate of morbidity and difficulties are associated with the surgical approaches of the craniovertebral junction (CVJ) [2,6]. Wanebo and Chicoine [7] stated that the resection of tumors of this region remains a technically demanding surgical procedure. The shape and size of the FM play an important role in the pathophysiology of various disorders of the CVJ [8]. Variations in the shape of the FM are significant in clinical, radiological, and surgical aspects [5,9]. According to Natsis et al. [10], the shape of the FM affects the surgical area and stated that around the FM provides a wider operative angle than an oval or rhomboid one. Similarly, Muthukumar et al. [11] stated that in the transcondylar approach, less bony resection will be performed in round FM compared to ovoid FM.

Additionally, the length, width, and area of the FM and its relationship with surrounding structures provide critical information for the surgical applications that require complete or partial resection of the CVJ [2]. Natsis et al. [10] reported that the long length and width of FM supply a wider operative ﬁeld and reduce the amount of bony extraction. Most of the previous studies [12-16] have reported that the length and width of the FM in females are smaller than the males. Therefore, it is believed that a surgical approach may be more difficult for females [17]. Furthermore, Landi et al. [18] stated that a wider FM decreases the potential risk of herniation due to postoperative hematomas or cerebellar swelling.

There are many pathologic conditions including intradural tumors such as neurinomas, meningiomas, and arteriovenous malformations of the vertebral artery and vertebrobasilar junction; extradural tumors such as chordomas, basilar invagination, and other congenital anomalies; rheumatoid arthritis and traumatic entities with atlantoaxial subluxation [2]. During transcondylar surgery, the application of the condylar resection in operations such as the excision of tumors is important for revealing this region. FM morphology and variations should be known for the execution of these applications [19]. The transoral, transmaxillary, transcondylar, and lateral cranial base approaches require well-controlled resection and reconstruction in the CVJ, which has a vital significance in the direct view of the lower cranial nerves, brainstem, and the vertebral artery [2]. Structures passing through the FM may be exposed to compression in cases such as achondroplasia and brain herniation [5].

Computed tomography (CT) is considered a good method for evaluating bony structures. Also, cone-beam computed tomography (CBCT) is an advantageous method due to its low radiation dose and cost [20,21]. This study aims to investigate the morphometric and morphological features of the FM in the Turkish population on the CBCT images and to review the literature in detail.

## Materials and methods

This retrospective study was approved by the ethics committee of Gaziantep University (approval date: 26/09/2018 and approval number: 2018/257). CBCT images of 400 individuals (200 males, 200 females, aged between 18 and 65) who were admitted to Gaziantep University Faculty of Dentistry for any reason were obtained by the Planmeca Promax 3D scanner on multiplanar sections in standard resolution mode, voxel size: 0.4 mm^3^ and 16×9, 16×16 cm^2^ FOV. Patients with any cranial or structural disorders, pathologic involvement of the CVJ, and history of the skull base trauma or pathologies that could affect the morphology or morphometry of the FM were excluded. The CBCT images were evaluated by Planmeca Romexis Viewer. The CBCT images were reviewed by two observers who were blinded to each other, and the final decisions were made after the consensus of the two observers. Discrepancies were resolved by a third observer.

The following six parameters were measured or calculated on these images: (i)* *Length of the FM (LFM): distance between basion and opisthion; (ii)* *Width of the FM (WFM)*:* distance between the two most lateral points on lateral borders of the FM; (iii)* *Area of the FM (AFM): it was evaluated through following three different methods:

Calculated by using Radinsky formula [22] (AFM-R) (=1/4*π*LFM*WFM)

Calculated by using Teixeira formula [23] (AFM-T) (=π*((LFM+WFM)/4)^2^)

Measured by using Planmeca Romexis Viewer (AFM-P)

(iv) The perimeter of the FM (PFM): it was measured by using Planmeca Romexis Viewer; ​​​​​​​(v) the shape of the FM (SFM): it was classified according to the shape of the FM; ​​​​​​​(vi) FM index (FMI): it was evaluated according to the Martin and Saller classiﬁcation [24] (=100*WFM/LFM; narrow: ≤81.9, medium: 82.0-85.9 and large: ≥86.0).

Statistical analysis

Data are normally distributed according to the Shapiro Wilk test, and Student t-test was used for the comparison of two groups of independent and normally distributed variables. Pearson correlation coefficient was used for testing the association between numerical variables. Mean±standard deviation for numerical variables, quantity, and % values for categorical variables are given as descriptive statistics. SPSS for the Windows version 22.0 package program was used for statistical analysis, and p<0.05 accepted as statistically significant.

## Results

The FM was examined in detail on the CBCT images of 200 females (mean age: 40.56±13.77) and 200 males (mean age 41.20±14.68) between the ages of 18 and 65. No significant age difference existed between the genders (p=0.651).

Values of the FM measurements

The mean values of the LFM, WFM, PFM, AFM-R, AFM-T, and AFM-P were found to be 36.75±2.50 mm, 32.55±2.93 mm, 108.35±7.50 mm, 941.81±128.26 mm^2^, 946.83±127.39 mm^2^, and 895.76±123.50 mm^2^, respectively. There was a statistically significant difference between the genders in terms of LFM, WFM, PFM, AFM-R, AFM-T, and AFM-P values (Table 1).

**Table 1:** The measurements of the foramen magnum ^α^Significant difference, *measured by using Planmeca Romexis Viewer, **by using Radinsky formula, ***by using Teixeira formula [23]. SD: standard deviation, M: male, F: female, T: total, LFM: length of the foramen magnum, WFM: width of the foramen magnum, AFM: area of the foramen magnum, PFM: perimeter of the foramen magnum

Parameter	T (mean±SD)	M (mean±SD)	F (mean±SD)	p-value
LFM (mm)*	36.75±2.50	37.66±2.40	35.84±2.26	0.001^α^
WFM (mm)*	32.55±2.93	33.39±2.99	31.72±2.62	0.001^ α^
PFM (mm)*	108.35±7.50	110.78±7.02	105.92±7.19	0.001^ α^
AFM-R (mm^2^)**	941.81±128.26	989.38±126.80	894.25±111.15	0.001^ α^
AFM-T (mm^2^)***	946.83±127.39	994.71±125.44	898.94±110.42	0.001^ α^
AFM-P (mm^2^)*	895.76±123.50	944.56±117.75	846.95±109.22	0.001^ α^

**Figure 1:**
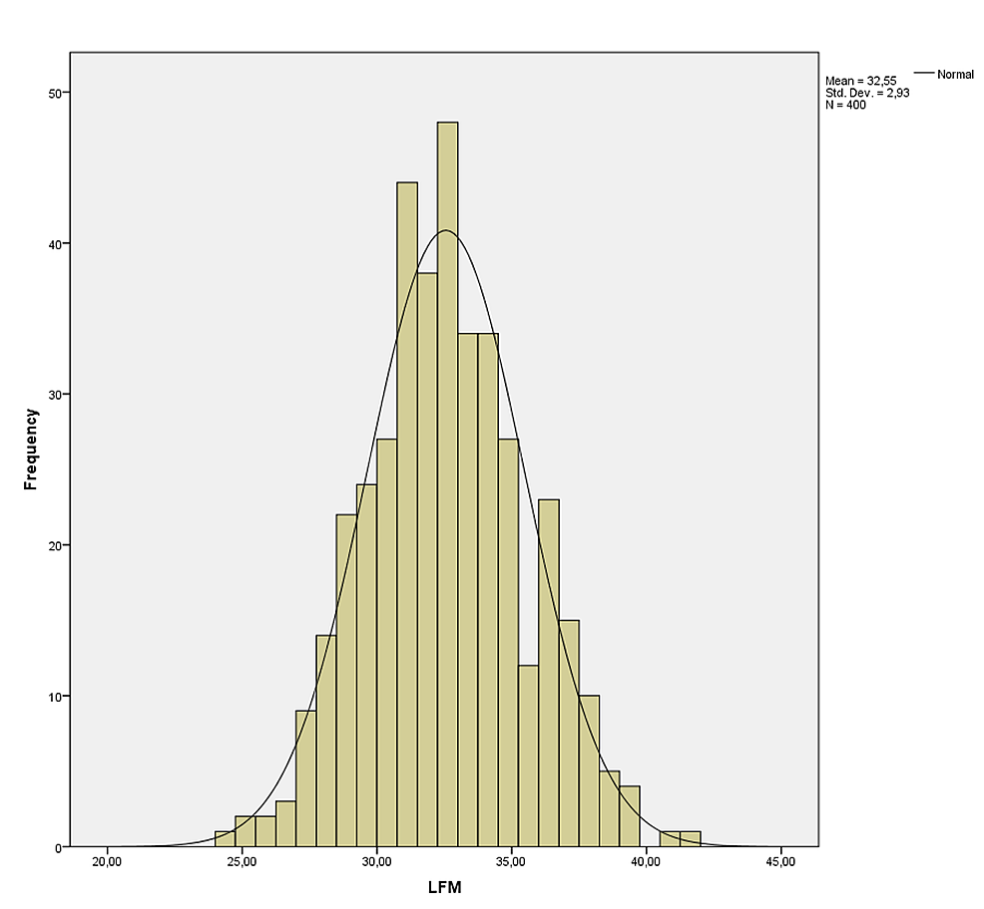
Histogram of distribution of the length of the foramen magnum

**Figure 2:**
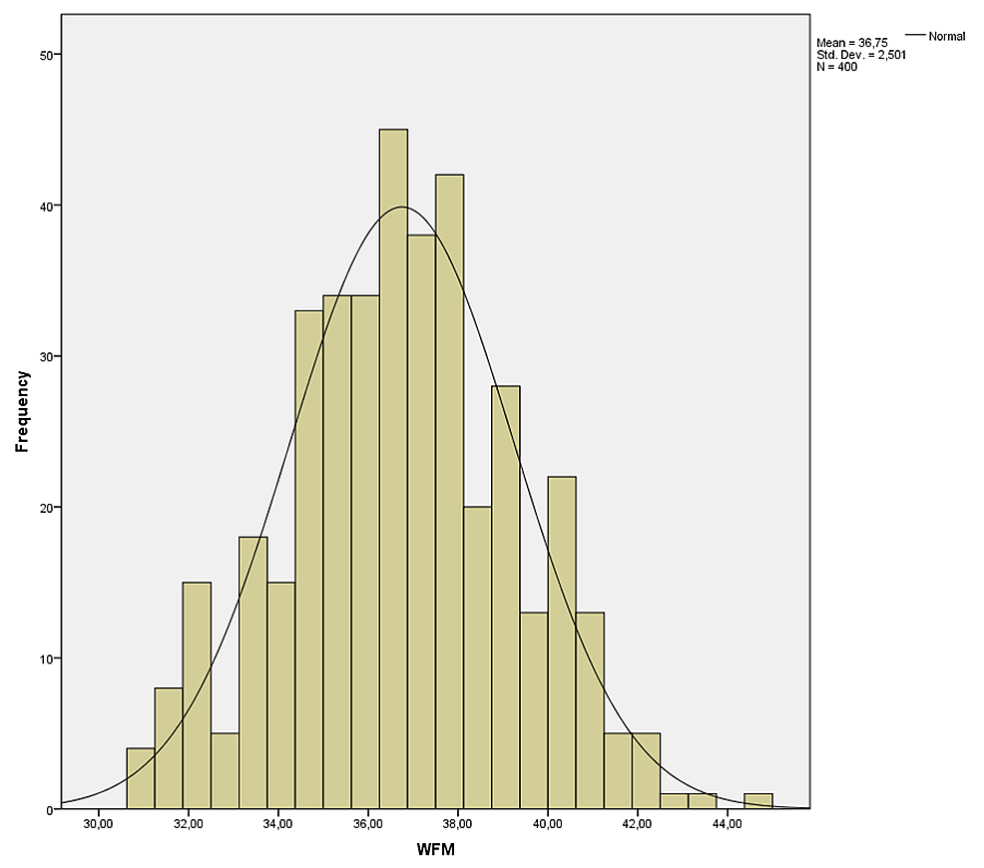
Histogram of distribution of the width of the foramen magnum

**Figure 3:**
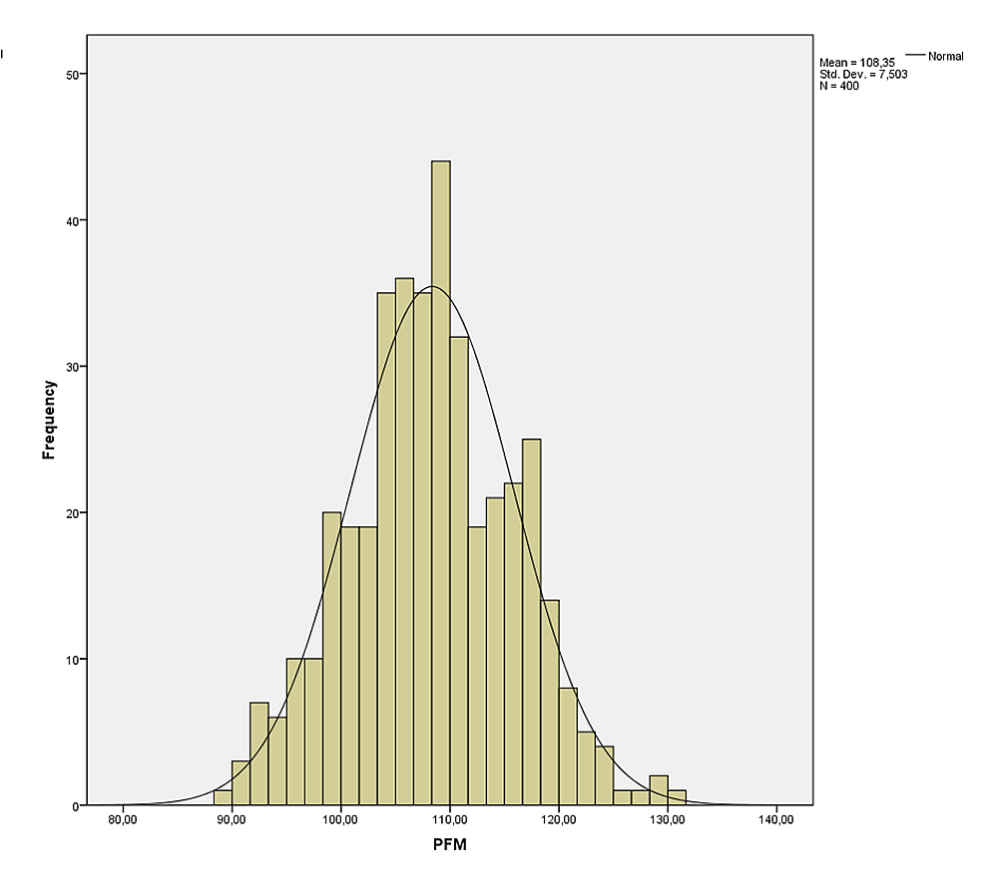
Histogram of distribution of the perimeter of the foramen magnum

There was no correlation between age and these values (p=0.680, p=0.945, p=0.546 p=0.738, p=0.768 and p=0.799, respectively). AFM-T was statistically higher than AFM-R and AFM-P (p=0.001 and p=0.001), and AFM-R was statistically higher than AFM-P (p=0.001). It was shown on a histogram of the distribution of the LFM in Figure 1, WFM in Figure 2, and PFM in Figure 3.

The shape of the FM

**Figure 4:**
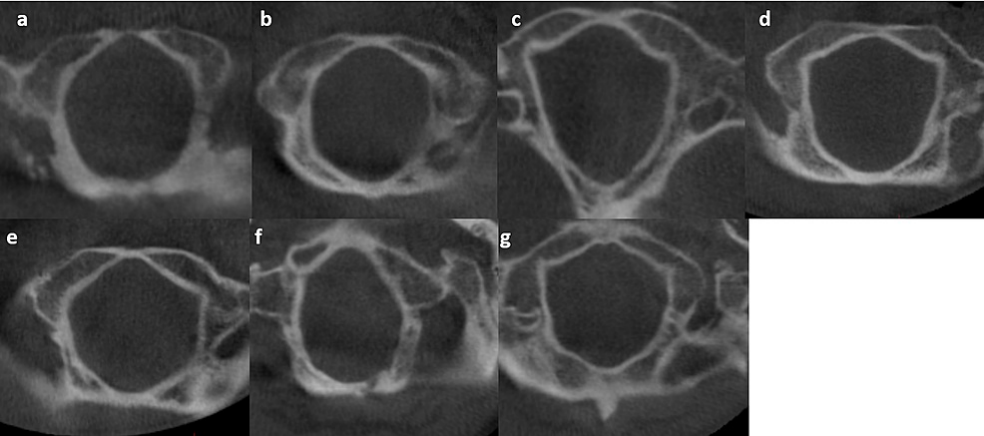
Shapes of the foramen magnum (a) oval, (b) round, (c) tetragonal, (d) pentagonal, (e) hexagonal, (f) egg, and (g) irregular

Seven different shapes of the FM were observed as oval, round, tetragonal, pentagonal, hexagonal, egg, and irregular (Table 2 and Figure 4). The most commonly observed shape of FM was oval and the least commonly observed shape was the egg shape in both genders. It was found that gender had no effect on the SFM (p=0.40).

**Table 2:** Values of the shapes of the foramen magnum M: male, F: female, T: total

Shape	M (n/%)	F (n/%)	T (n/%)
Oval	48 (24%)	52 (26%)	100 (25%)
Round	29 (14.5%)	26 (13%)	55 (13.75%)
Tetragonal	29 (14.5%)	23 (11.5%)	52 (13%)
Pentagonal	34 (17%)	37 (18.5%)	71 (17.75%)
Hexagonal	35 (17.5%)	42 (21%)	77 (19.25%)
Egg	5 (2.5%)	4 (2%)	9 (2.25%)
Irregular	20 (10%)	16 (8%)	36 (9%)
Total	200 (100%)	200 (100%)	400 (100%)

FM index

**Figure 5:**
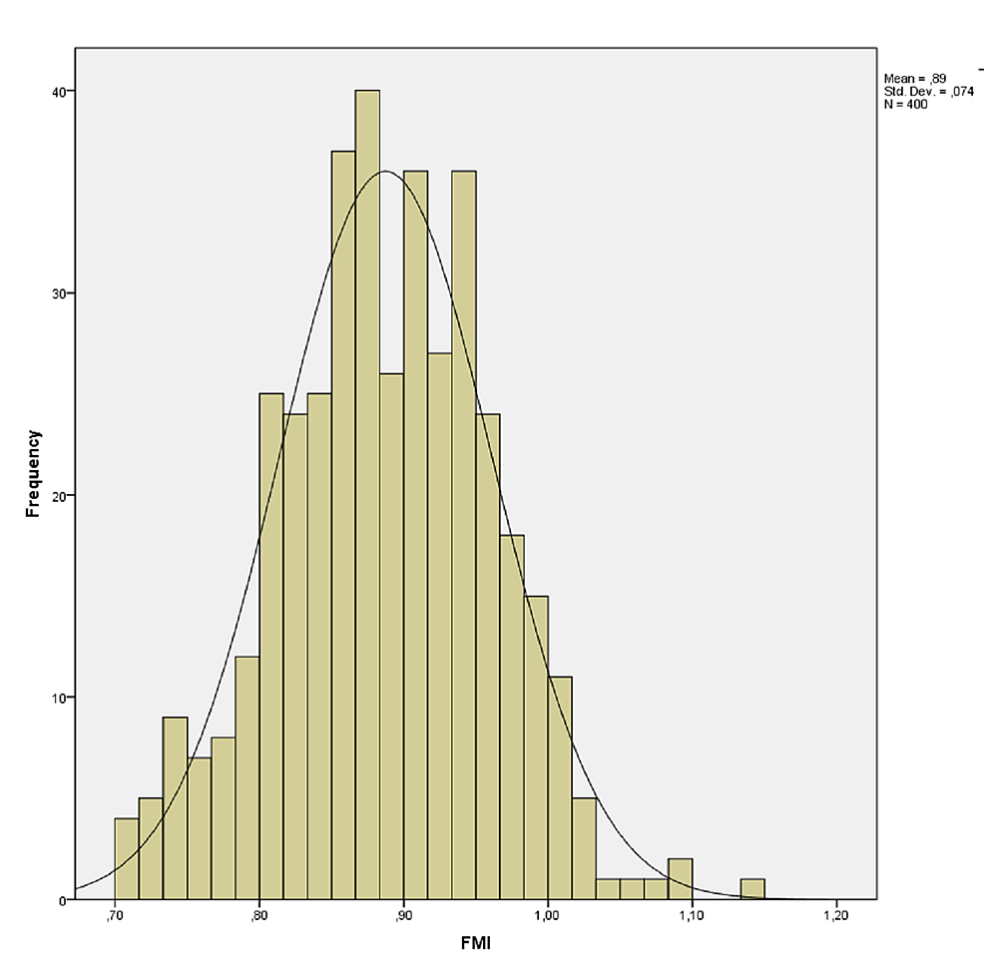
Histogram of distribution of the foramen magnum index

FM was classified according to the Martin and Saller classiﬁcation [24]. The mean of the FMI was found as 0.89±0.07 (0.71-1.14; Figure 5). It was found that 16.5% of the cases belonged to the narrow, 16% belonged to the medium, and 67.5% to the large index. No significant mean FMI existed between the genders (p=0.827). There was no correlation between age and FMI (p=0.726).

## Discussion

Zdilla et al. [3] stated that the anatomy of the FM was examined in many interdisciplinary studies such as comparative anatomy, forensic anthropology, physical anthropology, evolutionary biology, and surgery. In these studies, the anatomy of the FM was examined for many reasons, such as age determination [5], sex determination [5,25-27], identification of fire victims [28], and evaluation of surgical approaches at the CVJ [7]. In addition, FM has also been studied in other organisms [29]. Nevell and Wood [29] stated that some of the osteological features in the skull like FM might have undergone evolutionary changes.

The irregular shape of the FM is emphasized by the developmental anomalies of the soft tissues and the bones at the CVJ [4]. The FM is a basic element of the complex interaction of ligamentous, muscular, and bony structures constituting the CVJ. The size and shape of the FM are critical parameters for the indication of symptoms and clinical signs in CVJ pathologies. These pathologies involve signs and symptoms attributable to vascular compromise, motor myelopathy, sensory abnormalities, brainstem, and lower cranial nerve dysfunctions [8]. Diseases related to anomalies of the FM include occipital vertebra, condylar hypoplasia, atlas assimilation, and basilar invagination [30]. Besides these, knowledge regarding the anatomy of the FM plays a crucial role in the anterior vertebral artery mobilization, intradural extramedullary cervical spine tumors, occipital screw placement for occipitocervical fixation, transoral odontoidectomy for decompression of the basilar invagination, and the treatment of the atlantoaxial instability [31]. Excision of tumors around the FM remains a difficult surgical operation [7]. Morphological and morphometric evaluation of the FM before the surgical intervention to this region can be life-saving [2,17]. In addition, Chethan et al. [4] stated that these parameters have medico-legal importance and are useful for the identification of unknown individuals. Ukoha et al. [32] stated that sexual differences of the FM had been studied in various populations due to the importance of population-related variations in defining sexual differences.

From a clinical point of view, the morphology, morphometry, and variations of the FM can help with the diagnosis, classification, and treatment of diseases associated with FM anomalies [8]. For this reason, various studies have been conducted on the morphology and morphometry of the FM. These studies were performed on dry bones [2,4,8,10,13,25,26,33-39], cadavers [40], CT [9,17], CBCT [12,16,41], 3DCT [14,15,42], MDCT [43], MRI [44], radiography [45], and MSCT data acquired in the context of the virtopsy [27].

Given these facts, we believe that the morphological and morphometric evaluation of the FM and detailed comparison of obtained results with the previous studies will have important contributions to the literature.

Morphometrical analysis

Babu et al. [46] stated that lesions of CVJ pose a surgical challenge and are associated with high mortality, morbidity, and incomplete tumor removal. There may be many lesions in this area [2]. Many surgical approaches and various modifications have been developed to approach these lesions safely and effectively [10]. The type and size of the lesion determine which technique to use [2]. During these procedures, information about the morphometry, morphology, and variations of the FM may affect the surgical outcome [10]. Tubbs et al. [8] stated that physicians who make diagnoses regarding this area and perform surgical treatment of this region should know the morphometric features of the FM. Govsa et al. [2] stated that the measurements of the FM gain importance in the determination of the necessary resection thus reaching the lower clivus and premedullary region in the transcondylar aspect. It was reported that the area of the FM depends on both the perimeter and the length of the FM. Additionally, a surgeon can opt for a partial resection due to the expansion of the perimeter size. It should be noted that the biomechanical and anatomic results of partial resection on a narrow index differ from those in a wider index of FM. The same amount of partial resection may lead to a greater occipitocervical instability in FMs with longer perimeter while a more extensive resection is needed for optimum visualization in shorter perimeters. The surgical considerations involving the partial resection and reconstruction of FM are affected primarily by the PFM and secondarily by the LFM [2].

In joint-sparing transcondylar resection, not every patient is eligible for the application of the same procedure [47]. Recommendations for the degree of occipital condyle removal vary widely, ranging from no resection to complete resection [48]. Kamal et al. [47] stated that this variability is largely due to individual anatomical differences between patients and their preexisting pathology. Therefore, prior to the surgical procedure, the preoperative CT images of the skull base must be evaluated.

On the other hand, LFM and WFM differ in many diseases compared to healthy ones. Aydin et al. [49] stated that LFM was significantly larger in Chiari type I malformations in adults than in the control group. Similarly, Bliesener and Schmidt [45] stated that children with Arnold-Chiari malformation had significantly greater WFM than the healthy group.

Most of the previous studies [10,38,41-44] have found that males have significantly higher LFM and WFM diameters than females. There are also many studies that have reported that these measurements can be used in gender determination in forensic medicine [27]. Natsis et al. [10] stated that large LFM and WFM offer a wider operative ﬁeld and reduce the amount of bony extraction. Göçmez et al. [17] stated that a surgical approach for females may be more difficult because of this difference between the genders. Furthermore, Landi et al. [18] stated that a wider opening in the AFM reduces the potential risk of herniation due to postoperative hematomas or cerebellar swelling. For the far-lateral approach, it should be studied carefully to evaluate the features of the lesion, its neighboring vasculature, and the bony anatomy of the FM, occipital condyles, and jugular tubercles [47].

There are many studies that have investigated LFM and WFM because of their clinical significance (Table 3) [2,4,8,10,12-16,25-27,33-39,41-44,49]. In the previous studies, the average values of the LFM were reported as having a range of 31.72±2.14 to 36.66±2.26 mm in females and 34.04±2.36 to 38.17±2.70 mm in males and the average values of the WFM were reported as having a range of 26.31±1.15 to 31.09±2.36 mm in females and 28.63±1.89 to 32.98±2.78 mm in males, similar to our results.

**Table 3:** Measurements of the LFM and WFM, and comparison with the literature *Significant difference, M: male, F: female, T: total, CT: computed tomography, CBCT: cone-beam computed tomography, MDCT: multidetector CT, MSCT: multi-slice CT, LFM: length of the foramen magnum, WFM: width of the foramen magnum

Study	Race	Specimen	Method	n		Age		LFM (mm)		WFM (mm)	
				F	M	F	M	F	M	F	M
Aydin et al. [49]		Alive	MRI	15	15	52.2±18.2		25.2±3.8			
Lakshmi [44]		Alive	MRI	164	273	≥15		34.87±3.04*	36.52±3.25*		
Madadin et al. [43]	Saudi Arabian	Alive	MDCT	100	100	41.73±13.08	35.23±10.55	36.10±2.65*	37.21±2.15*	30.60±2.47*	31.65±2.25*
Tambawala et al. [41]	Indian	Alive	CBCT	115	111			34.46±2.35*	36.22±2.33*	29.16±2.53*	30.80±2.51*
Edwards et al. [27]	Swiss	Virtopsy	MSCT	106	144	56.8±18.39	49.6±16.12	36.66±2.26	38.17±2.70	31.34±2.19	33.05±2.61
Sukumar et al. [42]	Indian	Alive	CT	32	22	45.66±18.84	46.66±14.56	31.77±2.05*	35.18±2.84*	26.31±1.15*	29.53±2.76*
İlgüy et al. [16]	European descent		CBCT	95	66	45.66±16.95		35.62±2.43*	37.79±2.25*	31.09±2.36*	32.69±2.29*
Govsa et al. [2]	Turkish	Dry skull	Photographs	352				37.2±3.5		30.8±2.9	
Tubbs et al. [8]		Dry skull	Photographs	32	40	50-90		31 (25-37)		27 (24-35)	
Tellioglu et al. [15]	Turkish	Alive	3DCT	50	50	19-88		32.99±2.65*	34.73±2.21*	28.4±2.72*	30.47±2.25*
Burdan et al. [14]	Eastern European		3DCT	171	142	24.17±2.78	24.53±2.99	35.47±2.60*	37.06±3.07*	30.95±2.71*	32.98±2.78*
Gocmen Mas et al. [39]		Dry skull	Caliper	150				34.38±2.38		28.95±2.19	
Catalina-Herrera [38]		Dry skull	Caliper	26	74			34.3±0.4*	36.2±0.3*	29.6±0.3*	31.1±0.3*
Naqshi et al. [37]	Indian	Dry skull	Caliper	25				31.6±2.16		26.5±2.12	
Kumar et al. [36]		Dry skull	Caliper	17	19			33.22±2.00	36.78±1.52	29.49±1.66	30.05±2.36
Chethan et al. [4]		Dry skull	Caliper	53				31±2.4		25.2±2.4	
Lyrtzis et al. [13]	Greek	Dry skull	Caliper	68	73			33.86±2.31*	36.16±2.29*	28.97±2.32*	31.32±2.51*
Loyal et al. [35]	Kenyan	Dry skull	Caliper	64	138			35.0+7		38.5+6.5	
Natsis et al. [10]	Greek	Dry skull	Caliper	66	77			34.79±2.39*	36.20±3.39*	29.61±2.08*	30.92±3.15*
Radhakrishna et al. [26]	South Indian	Dry skull	Caliper	45	55			31.72±2.14*	34.04±2.36*	26.59±1.64*	28.63±1.89*
Ilhan et al. [34]	Turkish	Dry skull	Caliper	100				35.17±2.94		29.73±2.53	
Jain et al. [25]		Dry skull	Caliper	70	70			34.0±0.3	36.2±3.0	28.3±0.2	31.3±0.2
Kizilkanat et al. [33]	Turkish	Dry skull	Caliper	59				34.8±2.2		29.6±2.4	
Akay et al. [12]	Turkish	Alive	CBCT	102	88	46.8±15.7		34.66±2.31*	36.43±2.32*	29.78±2.05*	31.26±2.41*
Present Study	Turkish	Alive	CBCT	200	200	40.56±13.77	41.20±14.68	35.84±2.26*	37.66±2.40*	31.72±2.62*	33.39±2.99*

In addition, the AFM is also clinically important. Therefore, AFM has also been studied in many studies (Table 4) [2,8,13-16,25,27,36-39,41-43]. In these studies, it is seen that AFM is calculated by the Radinsky formula and/or Teixeira formula, and measured by using various different software. In order to make a comparison, all three methods were used in our study. Gocmen Mas et al. [39] stated that there were significant differences in between the mean AFM obtained from each of these three methods, similar to our results.

**Table 4:** Measurements of the AFM-R, AFM-T, and AFM-P, and comparison with the literature *Significant difference, **Via Software, M: male, F: female, T: total, CT: computed tomography, CBCT: cone-beam computed tomography, MDCT: multidetector CT, MSCT: multi-slice CT, AFM: area of the foramen magnum

Study	Race	Specimen	Method	n		Age		AFM-R (mm^2^)		AFM-T (mm^2^)		AFM-P (mm^2^)**	
				F	M	F	M	F	M	F	M		
Madadin et al. [43]	Saudi Arabian	Alive	MDCT	100	100	41.73±13.08	35.23±10.55			869.80±122.75*	925.84±98.20*		
Tambawala et al. [41]	Indian	Alive	CBCT	115	111			791.245±106.135*	877.883±108.782*	797.893±105.526*	884.884±108.504*		
Edwards et al. [27]	Swiss	Virtopsy	MSCT	106	144	56.8±18.39	49.6±16.12					812.14+94.52	887.69+124.10
Sukumar et al. [42]	Indian	Alive	CT	32	22	45.66±18.84	46.66±14.56						
İlgüy et al. [16]	European descent		CBCT	95	66	45.66±16.95							
Govsa et al. [2]	Turkish	Dry skull	Photographs	352								829±137.7	
Tubbs et al. [8]		Dry skull	Photographs	32	40	50-90						558 (385–779)	
Tellioglu et al. [15]	Turkish	Alive	3DCT	50	50	19-88						727±90	817±109
Burdan et al. [14]	Eastern European		3DCT	171	142	24.17±2.78	24.53±2.99					781.57±93.74*	877.40±131.64*
Gocmen Mas et al. [39]		Dry skull	Caliper	150				783.66±99.34		790.47±99.86		748.06±100.19	
Catalina-Herrera [38]		Dry skull	Caliper	26	74					801±17.4*	888.4±13.9*		
Naqshi et al. [37]		Dry skull	Caliper	25				660±90					
Kumar et al. [36]		Dry skull	Caliper	17	19			776.87±68.51	876.88±88.83				
Lyrtzis et al. [13]	Greek	Dry skull		68	73							726.26±111.07*	826.44±118.53*
Jain et al. [25]		Dry skull	Caliper	70	70					775±120	909±150		
Present Study	Turkish	Alive	CBCT	200	200	40.56±13.77	41.20±14.68	894.25±111.15*	989.38±126.80*	898.94±110.42*	994.71±125.44*	846.95±109.22*	944.56±117.75*

As for the PFM, this parameter has been studied in many studies (Table 5) [2,12-16]. In these studies, PFM were reported as having a range of 102.21±6.88 to 113.08±9.09 mm in females and 107.94±6.46 to 119.82±10.23 mm in males, similar to our results.

**Table 5:** Measurements of the PFM, and comparison with the literature *Significant difference, M: male, F: female, T: total, CT: computed tomography, CBCT: cone-beam computed tomography, MDCT: multidetector CT, MSCT: multi-slice CT, PFM: perimeter of the foramen magnum

Study	Race	Specimen	Method	n		Age		PFM (mm)	
				F	M	F	M	F	M
İlgüy et al. [16]	European descent		CBCT	95	66	45.66±16.95		102.21±6.88*	108.10±7.11*
Govsa et al. [2]	Turkish	Dry skull	Photographs	352				115.6±9.9	
Tellioglu et al. [15]	Turkish	Alive	3DCT	50	50	19-88		113.08±9.09*	119.82±10.23*
Burdan et al. [14]	Eastern European		3DCT	171	142	24.17±2.78	24.53±2.99		
Lyrtzis et al. [13]	Greek	Dry skull		73	68			110.87±8.89*	118.40±8.39*
Akay et al. [12]	Turkish	Alive	CBCT	102	88	46.8±15.7		102.67±6.14*	107.94±6.46*
Present study	Turkish	Alive	CBCT	200	200	40.56±13.77	41.20±14.68	105.92±7.19*	110.78±7.02*

Additionally, the shape of the FM is also clinically important. Murshed et al. [9] stated that variation in the shape of FM should be taken into consideration during the clinical and radiological diagnostic procedures and the surgical approach. For this reason, it has been studied in many studies [2,10,14,26,27,34,35]. The shape of FM differs in many studies as opposed to the LFM, WFM, and PFM.

The most frequently observed FM type was reported as oval by Radhakrishna et al. [26] (39%), as tetragonal by Govsa et al. [2] (25.66%) and Ilhan et al. [34] (24%), as polygonal by Loyal et al. [35] (63%), and as round by Edwards et al. [27] (26%). In this study, similar to Radhakrishna et al. [26], the most frequently observed shape was oval with 25%.

FMI is also important for the choice of surgical approach [34]. The results of partial resection in a narrow index are different from those obtained in a wide FMI [2]. Additionally, as FMI increases, more extensive bone resection is required in surgeries [4].

The average value of the FMI was calculated as 1.2±0.1 by Chethan et al. [4], 1.19±0.09 by Ilhan et al. [34], 1.2±0.1 by Kizilkanat et al. [33], 0.85±0.06 by Madadin et al. [43], and 0.84±0.06 by Naqshi et al. [37]. In this study, it was found to be 0.89±0.07.

## Conclusions

Vital neurological structures which relay information to and from the brain and spinal cord pass via the foramen magnum. Morphometric and morphological features of the foramen magnum located in the craniovertebral junction, which is a highly complex area, are variable. Surgical procedures and approaches in this region are essential due to the high mortality rate. For this reason, anatomical structures in these regions should be well known before surgery. The quantitative data presented in this study, which made a detailed literature comparison, may assist in surgical procedures around the foramen magnum and the planning of these procedures.
